# A folliculocentric variant of extragenital lichen sclerosus presenting in a 29-year-old man

**DOI:** 10.1016/j.jdcr.2023.07.022

**Published:** 2023-07-27

**Authors:** Rachel Wetstone, Mary Awad, Kaitlyn Yim, Zendee Elaba, Nikki A. Levin

**Affiliations:** a Herbert Wertheim College of Medicine, Florida International University College of Medicine, Miami, Florida; b Department of Dermatology, University of Massachusetts, Chan Medical School, Worcester, Massachusetts; c Department of Pathology, University of Massachusetts, Chan Medical School, Worcester, Massachusetts

**Keywords:** autoimmune disease, clinical cases, comedo-like, connective tissue disease, dermoscopy, extragenital lichen sclerosus, follicle, follicular, folliculocentric, general dermatology, inflammation/inflammatory, lichen sclerosus

## Introduction

Lichen sclerosus (LS) is a chronic inflammatory skin condition characterized by hypopigmented atrophic macules, papules, patches, plaques, and erosions. About 85% of LS cases involve the anogenital region, while the remaining case are extragenital.^1^ Some patients present with both types of involvement. Extragenital LS is more common in women and typically occurs on the trunk or extremities. We present an unusual case of folliculocentric extragenital lichen sclerosus in a man and demonstrate the use of dermoscopy in helping to make this diagnosis.

## Case report

**Fig 1:**
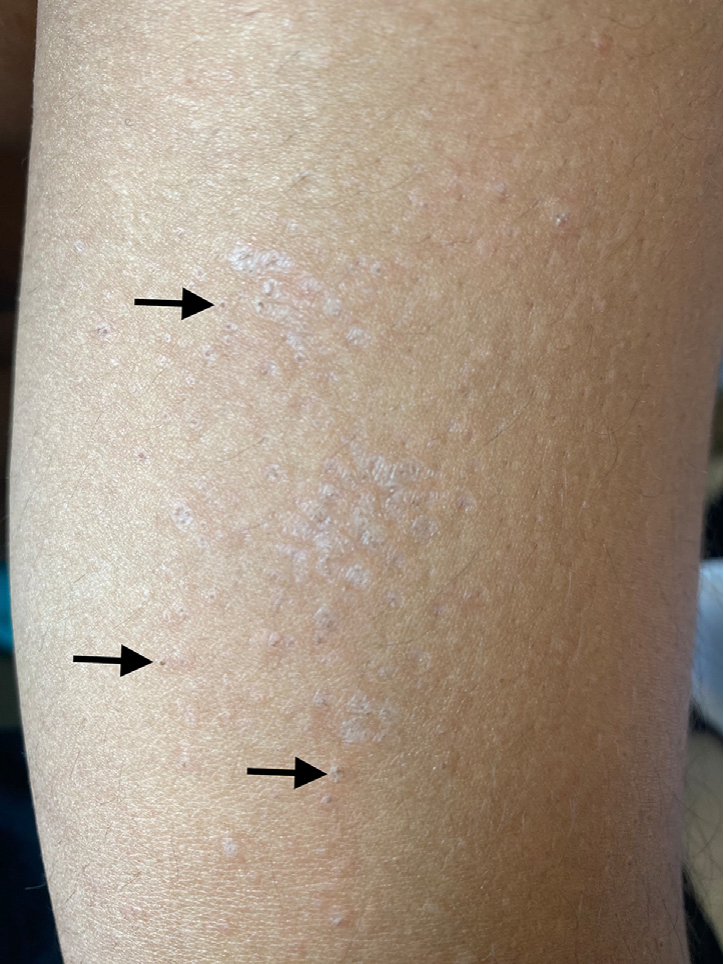
Volar forearm showing porcelain *white* hypopigmented atrophic macules coalescing into patches and some keratotic follicular plugs (*black arrows* show examples).

**Fig 2:**
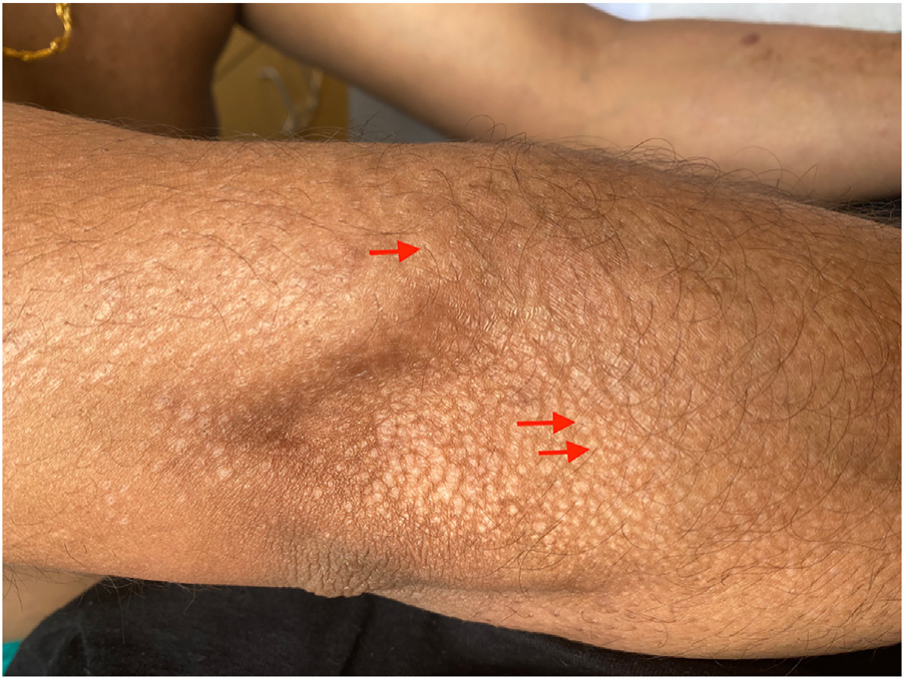
Dorsal forearm and elbow with hypopigmented atrophic macules coalescing into patches. Terminal hairs can be seen emanating from many of the lesions (*red arrows* show examples).

**Fig 3:**
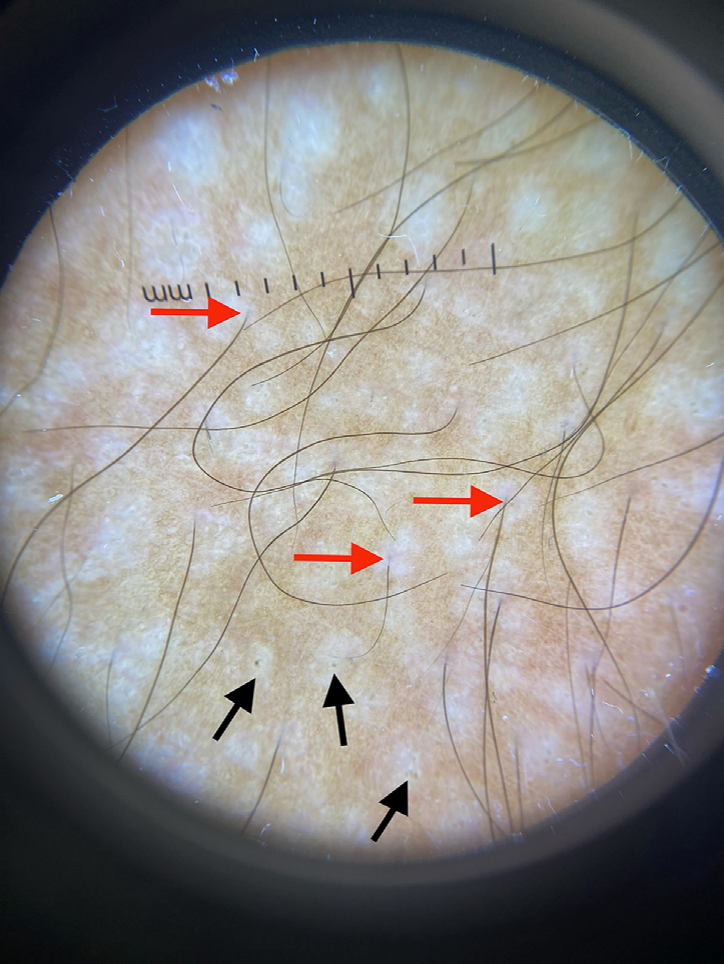
Dermoscopy of forearm lesions showing hypopigmented macules with terminal hairs (*red arrows* show example) and keratotic plugs (*black arrows* show example).

A 29-year-old man with no history of skin or autoimmune disease presented with a 6-months history of an intermittently itchy rash consisting of light colored macules that began on his wrists and then spread up his arms. On initial presentation, he was treating the rash with triamcinolone 0.05% cream twice daily from his primary care physician with no improvement after weeks of use. He was not taking any medications or supplements. Physical examination revealed hypopigmented atrophic coalescing macules with keratotic plugs reminiscent of open comedones on the bilateral volar forearms (Fig 1) extending onto the dorsal forearms (Fig 2), as well as a single patch on the sacrum. Many of the macules had central hair follicles. Dermoscopy revealed hypopigmented macules with keratotic plugs (Fig 3). The differential diagnosis included LS, morphea, discoid lupus, lichen planus, and anetoderma.

**Fig 4:**
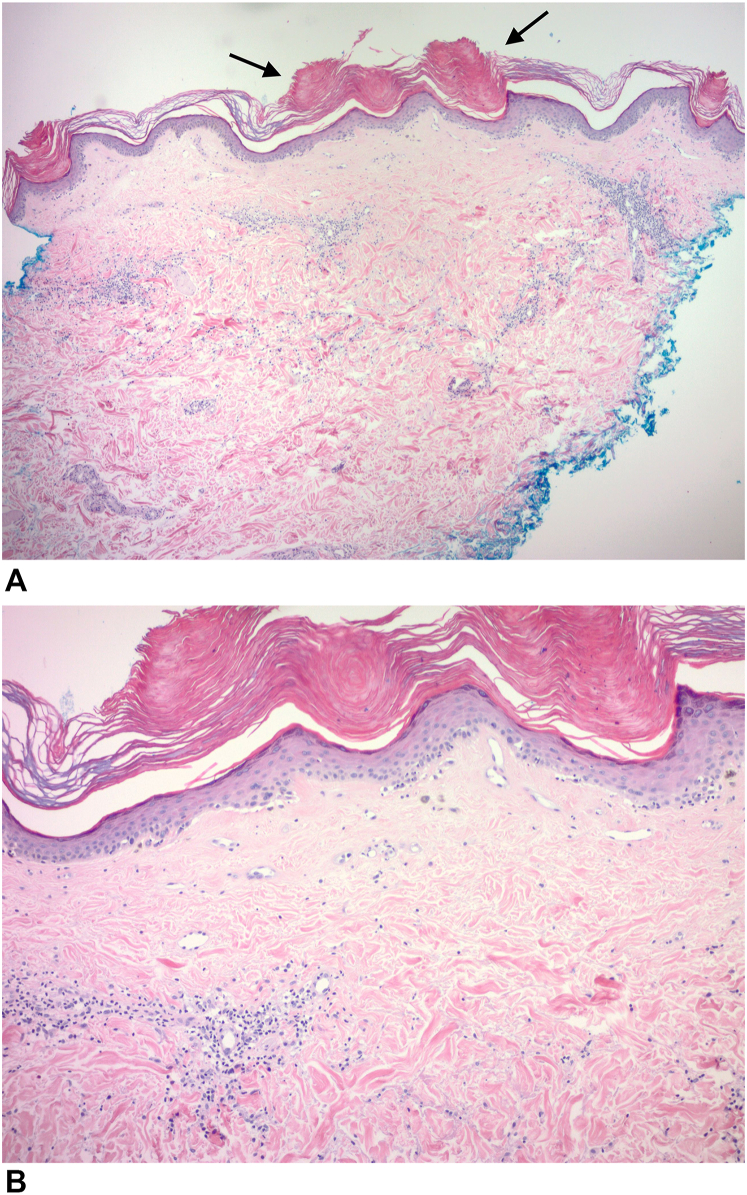
**A,** Low magnification showing epidermal atrophy, superficial dermal sclerosis, and follicular plugging (*black arrows*) (H&E ×40). **B,** Higher magnification showing mild vacuolar changes of the basilar epidermis, associated with homogenized collagen and melanophages in the superficial dermis. Perivascular lymphohistiocytic inflammation was present in the superficial to mid dermis (H&E ×100).

A 4-mm punch biopsy was performed on the left volar forearm. The specimen showed epidermal atrophy with mild vacuolar changes of the basal layer of the epidermis and follicular plugging. In the superficial dermis, there was sclerosis with homogenization of collagen and melanophages. Perivascular lymphohistiocytic inflammation was present in the superficial to mid dermis (Fig 4). These histologic findings were consistent with a diagnosis of lichen sclerosus. No other laboratory studies were performed. Taken together with the physical examination and dermoscopy findings, this patient’s presentation was most consistent with the folliculocentric variant of LS.

Treatment options including phototherapy and topical corticosteroids were discussed with the patient, and he was prescribed topical betamethasone dipropionate 0.05% ointment twice daily and recommended to start phototherapy. The patient was lost to follow up for 7 months, at which point he contacted the clinic with worsening lesions, not having started the topical corticosteroids or phototherapy. He was then started on narrow band ultraviolet B phototherapy 2-3 times weekly using the clinic’s standard psoriasis protocol for skin type IV. He was recommended to add alternating triamcinolone acetonide 0.1% ointment twice daily and tacrolimus 0.1% ointment topical twice daily to his treatment regimen along with the phototherapy. After 31 phototherapy treatments, he had marked improvement, and plans to continue treatment until the LS was cleared.

## Discussion

The prevalence of LS varies from 0.1% to 1.67%, with the extragenital form comprising only 15% to 20% of cases.^1^ Extragenital LS can occur simultaneously with genital LS; however, 6% of cases occur as isolated extragenital LS, as in this case.^1^ No consistent genetic inheritance pattern has been noted in LS, although there are reports of familial cases of LS with significant HLA class II antigen DQ7 association, suggesting genetic factors may be involved.^2^ There are currently no data on extragenital LS genetics.

Extragenital LS most commonly involves the trunk, proximal extremities, breasts, neck, shoulders, and buttocks.^3^ It typically presents as hypopigmented atrophic macules or patches, often with peripheral erythema and a central cigarette-paper-like appearance. Lesions with reticular striae and superficial ulceration have also been reported.^3^ Our case of extragenital LS is unusual in that it was folliculocentric, presented on the distal extremities, and had a plugged comedonal appearance on dermoscopy.

Dermoscopy can be a useful tool in the diagnosis of LS. Hypopigmented plaques with comedo-like openings in the center have been previously described as the dermoscopic pattern of extragenital LS.^4^ Histopathologically, these comedo-like openings correspond to dilated infundibula with cornified follicular plugging, though this finding can be seen in both genital and extragenital LS. Our patient had comedo-like openings and hairs at the center of several lesions apparent on physical examination (Figs 2 and 3), consistent with the diagnosis of folliculocentric extragenital LS. The folliculocentric variant of LS is characterized by shiny, hypopigmented macules with follicular scale and is distinguished from the classic presentation of extragenital LS mainly by its clinical presentation.^5^, ^6^, ^7^, ^8^ Previous case reports describing this uncommon clinical variant were all in females. Like prior cases, there was no evidence of direct follicular involvement or inflammation histologically. This case is an example of an unusual clinical presentation of extragenital LS in a man and highlights the utility of dermoscopy to aid in the diagnosis of this inflammatory skin condition.

## Conflicts of interest

None disclosed.
